# Media health literacy predicts preventive health behaviors: findings from a nationally matched survey

**DOI:** 10.3389/fdgth.2025.1659988

**Published:** 2025-09-30

**Authors:** Shadee Hall Ashtari, Daniela Rodrigues Recchia

**Affiliations:** ^1^ Department of Communication, Annenberg School of Communication and Journalism, University of Southern California, Los Angeles, CA, United States; ^2^ Institute for Research in Operative Medicine, Witten/Herdecke University, Witten, Germany; ^3^ Chair of Medical Theory, Integrative and Anthroposophic Medicine, Witten/Herdecke University, Witten, Germany

**Keywords:** media literacy, media health literacy, misinformation, social determinant of health, index development, health communication

## Abstract

**Objectives:**

To produce the first validated measurement of Adult Media Health Literacy (AMHL) and examine associations between scores on the new index and eight specific health behaviors and outcomes.

**Methods:**

A cross-sectional survey was conducted in 2023 with a non-probability sample of 589 U.S. adults ages 25–64, matched to national census demographics for age, gender, race, and education. The survey included the AMHL Index and outcome measures on smoking, vaping, vaccination, annual exams, mammography, colon cancer screening, and chronic health status. The predictive validity of the Index was evaluated using PLS-SEM. Covariate-adjusted linear and logistic regression models assessed the relationship between participants’ composite scores and reported health behaviors and outcomes.

**Results:**

All Index indicators demonstrated no collinearity concerns and a reliable measurement. Higher AMHL scores were significantly associated with higher odds of preventive health behaviors. A single-point increase on the Index was independently associated with increased odds of vaccination, mammography, and annual exam attendance, and decreased odds of smoking and vaping.

**Conclusions:**

The study provides the first validated AMHL measure and evidence for the independent role media literacy plays as a social determinant of health. Multi-sector intervention opportunities are discussed.

## Background

In a 2021 public health advisory, the U.S. Office of the Surgeon General declared health misinformation a “serious threat to public health” and its mitigation a “moral and civic imperative” (1). Issued during the COVID-19 pandemic, the advisory was responding to the rapid explosion of mis/disinformation* related to vaccines and digital media's role in its diffusion and validation. Four years later, the American public relies on digital media more than ever, with growing numbers citing the internet as their primary source of health information. Social media alone serves as a weekly source of health information for nearly a quarter of U.S. adults (2).

In a nationally representative survey conducted by Healthline and YouGov in 2024 (*N* = 4,012), more than half of American adults reported trying a new “health tactic”^†^ that they saw on social media in the last year. Results from an earlier large-scale survey, the 2022 Health Information National Trends Survey (HINTS) (*N* = 6,252), found that 36% of American social media users frequently encountered health misinformation and 67% reported difficulties identifying health misinformation (as measured via self-report questionnaire) (3). A study conducted by KFF the following year used an assessment-based estimation and found that 50%–75% of adults were unable to discern between false and true health claims (2).

As the National Academy of Sciences points out, “some of this misinformation is brain candy, simple entertainment, and inconsequential; some of it, though, has the potential to impact public health, inform policy responses, and shape people's perceptions of the world” (4). Across a wide range of health topics, a body of literature has shown that repeated and widespread exposure to health misinformation is linked to its growing acceptance and the formation of scientifically unfounded beliefs that inform critical personal, parental and community-wide health decisions (5–9). This extends beyond vaccination to countless other consequential health issues, including cancer care (10, 11). A 2024 analysis of the same HINTS data (*N* = 6,252) found that 10% of adults diagnosed with cancer have made fatal medical decisions—including rejecting evidence-based treatments such as surgery and radiation—based on information they saw on social media (9).

The broader implications are profound, with ripple effects that include preventable death and psychological suffering, the resurgence of eradicated diseases, and eroding public trust in civic and public health institutions. The latter has become especially salient in recent months as the National Institutes of Health (NIH), Centers for Disease Control and Prevention (CDC) and the Department of Health & Human Services have faced increasing politicization and escalating opposition to longstanding public health measures. As research continues to show upward trends of online health information-seeking as a decision aid, there is a pressing need for multi-level interventions that can curb the spread of false medical information and individuals’ susceptibility to it. This paper focuses on the latter.

### Adult media health literacy

Among other factors, susceptibility to health mis/disinformation has been independently linked to individuals’ levels of media literacy (ML)^‡^ and health literacy (HL). Health literacy interventions aim to reduce negative health outcomes associated with an inability to obtain, understand, or use essential health information and services. Most HL research focuses on older adults, people with chronic illnesses, and individuals with limited English proficiency. In contrast, ML research typically concentrates on adolescents, emphasizing critical inquiry, empowerment and self-reflexivity. Evidence shows that each construct is independently related to health-related beliefs and behaviors (12, 13). While each play an important role in informed decision-making, neither literacy alone accounts for the set of knowledge, abilities and practices (KAPs) that manifest at their intersection — that is, adult media health literacy.

As a sub-literacy within the overarching domain of ML, adult media health literacy (AMHL) represents a cumulative ability to: (1) *access* health-related information from various sources; (2) *identify* key elements of message construction; (3) *critically evaluate* message credibility, quality and relevance; (4) *produce* content using a variety of media tools; and (5) *engage* with a global media culture (76). Each of these is reinforced by specific KAPs, outlined in Supplementary Material 2, that fluctuate throughout the decades-long course of adulthood.

### Gaps in knowledge

The available body of MHL scholarship reveals four major limitations. The first is conceptual, with little known in regard to how AMHL manifests and whether or how it is linked to health beliefs, behaviors and outcomes. As reported in a preceding scoping review, the vast majority of MHL studies do not reference an explicit theory or model and those that do lack a formal representation or discussion of their framework (76).

Likely stemming from an insufficient theoretical understanding of how ML operates in health contexts, what research exists at the intersection of adult ML and health misinformation tends to focus on more politically charged issues, such as COVID-19 vaccination. Media literacy studies that examine susceptibility to science misinformation, for example, generally account for partisanship or critical reasoning capacities but tend to neglect the unique considerations associated with health behaviors. These can include barriers to high-quality health care and health information; the higher stakes of bodily decision-making; the disproportionate influence of anecdotal evidence; and the role of situational literacy (i.e., how stress can temporarily impair one's cognitive processing) (14). A review of the literature on individual susceptibility to mis/disinformation reveals that most studies focus on a small number of prominent political issues, such as gun control and climate change. As others have pointed out, the findings from these contexts are not necessarily generalizable to health and medicine—hence the need for intersectional constructs such as AMHL (15–19).

The second area in need of greater refinement is methodological. No AMHL study to date has been conducted with an instrument that employs direct measurement (i.e., performance based). Existing instruments for adults measure ML and HL separately, failing to capture a conceptual overlap, and are overly dependent on self-assessment measures. Significant issues of validity associated with self-report questionnaires for knowledge and skills assessments have been well documented (20–25). Yet, they remain prominent due to the burden of analyzing open-ended data, challenges of scale, and the design costs of task- and performance-based measures.

The third issue pertains to sampling. To date, no AMHL studies have been conducted with a representative sample of adults and what adult studies exist largely under-represent working-age adults, generally classified in the U.S. as those between the ages of 25 and 54. This period marks a time when adults are in their critical preventive health care years and most likely to care for children and/or elderly dependents, meaning they not only make consequential decisions for themselves, but also on behalf of their loved ones. The affordances and implications of AMHL among this demographic of health decision-makers are profound and span generations.

Finally, there is a clear need for the inclusion of diverse outcome variables that explore relationships beyond saturated health topics in health misinformation studies, such as vaccination. While there is a great deal of research dedicated to vaccine hesitancy, relatively little is known as it pertains to adult ML and other health topics associated with high levels of digital misinformation. Examples include misinformation on smoking products, diet, sunscreen, reproductive care, and mental health (5, 11, 26–28). Given that chronic non-communicable diseases account for eight of the 10 top causes of death in the U.S., further attention is warranted.

Cigarette smoking, for example, remains the leading cause of preventable disease. In 2022, roughly 12% of adults reported smoking cigarettes and 6.5% reported vaping, with rates rising (29, 30). Breast cancer affects one in eight American women and despite the life-saving role of screenings, recent estimates indicate that half of women aged 45–54 have not received a recommend mammogram under current guidelines (31). Colon cancer is the second most common cause of cancer deaths among all adults, yet roughly 30% of those aged 50–75 have not received recommended screenings (32). Lastly, while yearly medical exams for younger healthier adults are generally deemed unnecessary (33), annual visits are recommended for those 40 and older and have been shown to improve health outcomes and reduce mortality through preventive care screenings and interventions. As of 2020, only 5.3% of adults age 35 and older received all high-priority clinical preventive services, including but not limited to screenings for breast, colon, lung and cervical cancer (34). This marks a 5% decrease from 2015.

A wide range of socio-ecological contributors have been documented as predictors of both preventive health care engagement and risky health behaviors (35), though the role of contemporary factors such as ML have remained largely unexplored.

## Objectives

To narrow the gaps identified, the current study utilizes a socio-ecological framework to investigate whether ML operates as a social determinant of health. Toward this end, it was hypothesized that scores on a performance-based AMHL Index would independently predict behaviors associated with preventable^§^ health conditions, both acute (e.g., influenza) and chronic (e.g., cancer):

**Hypothesis 1:** Higher AMHL is associated with greater likelihood of flu vaccination (H1a); COVID-19 vaccination (H1b); receiving recommended mammograms (H1c); colon-cancer screenings (H1d); and attending annual physical exams (H1e).

**Hypothesis 2:** Lower AMHL is associated with greater likelihood of engaging in unhealthy behaviors linked to preventable chronic conditions: smoking (H2a) and vaping (H2b).

**Hypothesis 3:** Lower AMHL is associated with greater likelihood of reporting a chronic health condition.

## Theoretical framework

### Media literacy as a social determinant of health: A socio-ecological framework

The hypotheses are grounded in a socio-ecological model of health (SEM), which contends that human development is shaped by a series of interconnected variables across five levels: the individual, social, cultural, political, economic and chronological (36).^‖^ These variables are often collectively referred to as social determinants of health (SDH), defined by the World Health Organization as the *nonmedical factors* that shape human health, such as the “conditions in which people are born, grow, work, live, and age, and the wider set of forces […] shaping the conditions of daily life,” including social norms, policies, and economic systems (37).

**Figure 1:**
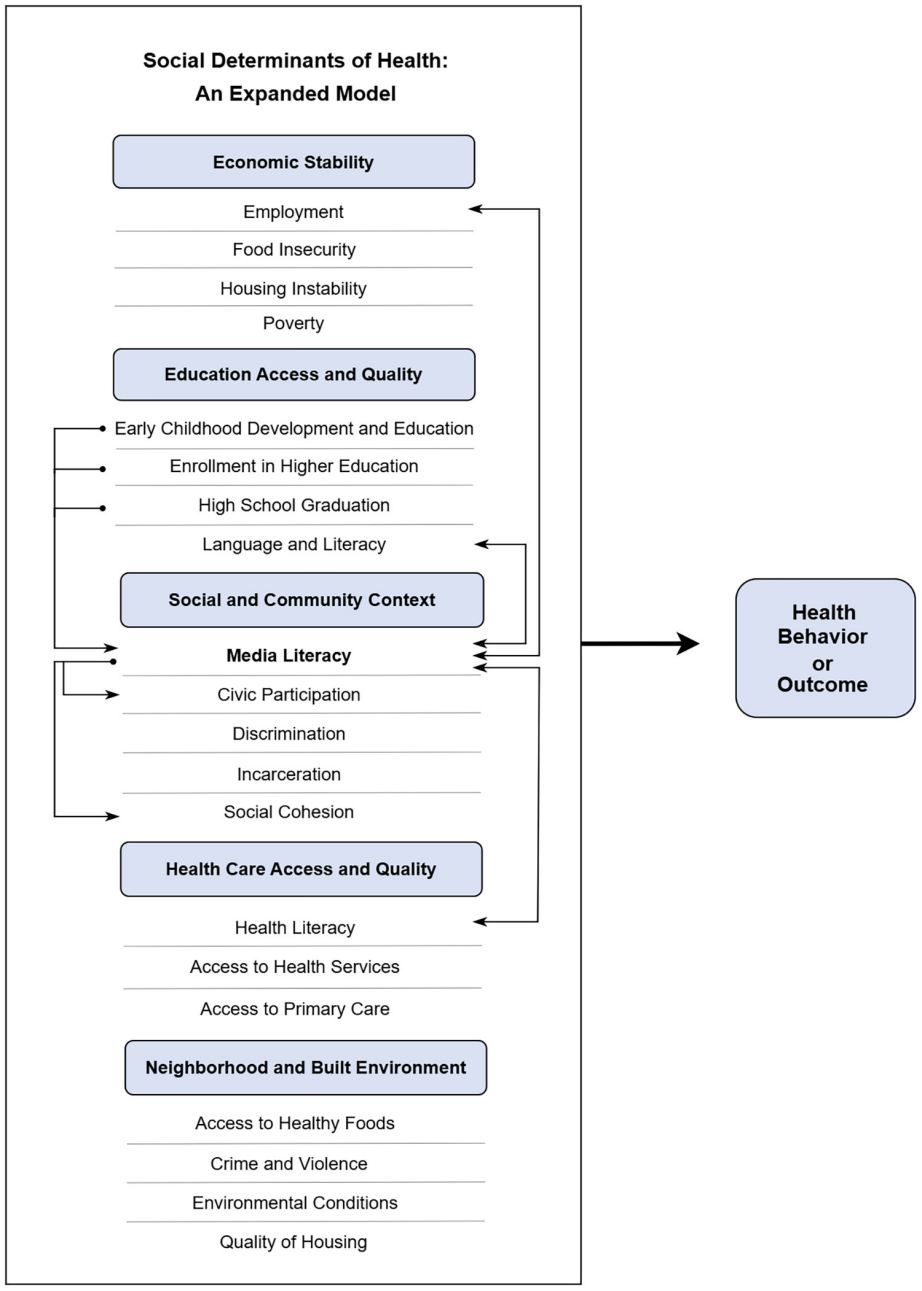
Media literacy as a social determinant of health. Researchers and practitioners are encouraged to substitute the umbrella construct of ML with more tailored sub-literacies depending on their specific population and subject of interest. Reproduced with permission from “Media literacy as a social determinant of health” by Shadee Ashtari, licensed under CC BY-NC-ND.

As illustrated in Figure 1 and Supplementary Material 1, the SDH are organized under five overarching categories and operate as an interdependent system (38). This means that modifying one variable can have potential domino effects. For example, while the SDH of “access to primary care health” is empirically linked with vaccination, other SDH such as higher education, employment, discrimination and ML moderate the relationship. To date, uni- and bi-directional relationships have been documented between ML and eight standing SDH variables: (1) early childhood development and education; (2) enrollment in higher education; (3) high school graduation; (4) health literacy; (5) language and literacy; (6) employment; (7) social cohesion; and (8) civic participation. As shown in the expanded model, ML operates as the 20th SDH within the Social and Community Context block. For a more comprehensive overview of the supporting evidence for this model, see Ashtari (76, pp. 1238–1241).

## Methods

Testing the hypotheses required a measurement of the predictor variable (AMHL). Given the lack of such an instrument, the first step involved developing a validated measurement that captured the full breadth of the construct's component KAPs.

### AMHL Index

**Figure 2:**
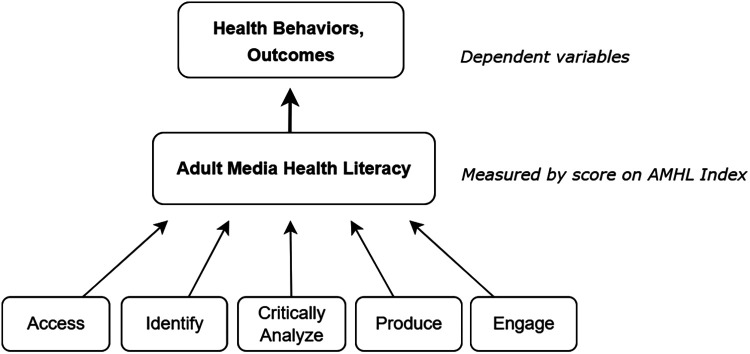
Formative model of AMHL Index.

In this study, AMHL represents a formative construct, meaning causality flows from the Index items to the construct (Figure 2). Hence, the Index comprises a census of items that serve as an explanatory combination of unequally weighted indicators (39). An initial battery of 45 performance items was developed based on the 17 evidence-based KAPs listed in Supplementary Material 2. Each item was scored using a companion rubric that yielded a composite AMHL score. Questions and Index scoring are detailed in Supplementary Material 3.

The following section describes the development and validation of the Index, which reflects best practices established by experts in index methodology (40–48).

### Face, content and construct validation

The draft instrument and scoring rubric were first reviewed by five subject-matter experts in survey methodology, health communication, ML, and quantitative and qualitative methods. Their feedback validated the Index's theoretical structure (Figure 2). Next, pre-testing was undertaken with a convenience sample of English-proficient adults aged 22–65 (*N* = 75). Participants submitted anonymous feedback on question wording, as well as the survey's duration, user friendliness, and technical accessibility.

Cognitive interviews were then conducted to reduce measurement error by optimizing question comprehension and response completeness. Following think-aloud protocols, eight hours of one-on-one Zoom interviews were conducted with six working-age adults who were compensated with $20 Amazon gift cards (49). They completed the survey aloud, explaining their thought process, rationale and hesitation around sensitive questions. As a result, select items were removed or reframed to reduce cognitive shortcuts and minimize social desirability effects. The total item count was reduced from 45 to 28.

A second pilot was conducted in March 2023 with 53 target population members who were recruited through Amazon Mechanical Turk and compensated $7. They completed an anonymous survey that included the Index and a slate of health outcome measures. Closed-ended questions were accompanied by required open-ended fields where participants explained how they interpreted questions, analyzed images and videos, and formed their answers. The qualitative data were manually assessed alongside their corresponding closed-ended questions as a cross-validation technique. The item count was reduced from 28 to 21 and the scoring rubric refined and finalized.

### External validation

The final step involved assessing the AMHL Index's external nomological validity, or the extent to which the measure meaningfully correlates with the theoretically-related outcome variables posited in the hypotheses. Toward this end, the final survey was fielded as part of a larger correlational study. Assuming a 5% alpha, 80% power and one predictor variable (Index score), a minimum sample size of 568 was required as calculated by G-Power statistical software. A non-probability sample of U.S. adults was recruited through Prolific to match national census data for distributions on age, gender, race, and education (50, 51–55). Sociodemographic characteristics are displayed in Table 1.

**Table 1:** Characteristics of participants: July–December 2023.

Characteristic	Full sample (*N* = 589)	
	*n*	%
Age		
25–29 years	94	16%
30–34 years	134	22.8%
35–39 years	82	13.9%
40–44 years	42	7.1%
45–49 years	67	11.4%
50–54 years	73	12.4%
55–59 years	55	9.3%
60–64 years	42	7.1%
Gender		
Woman	291	49.4%
Man	284	48.2%
Other	14	2.4%
Race		
Black or African American	83	14.1%
White	452	76.7%
Asian	34	5.8%
American Indian or Alaska Native	20	3.4%
Highest educational level		
No four-year degree	353	59.9%
Bachelor's degree	155	26.3%
Master's degree	62	10.5%
Doctorate	19	3.2%
Political affiliation		
Identify as democrat or lean toward democrat	296	50.3%
Identify as republican or lean toward republican	124	21.1%
Identify as politically independent	111	18.8%
No political affiliation	58	9.8%
Household income		
Low income ($0-$62,999)	317	53.8%
Middle income ($63,000-$188,999)	251	42.6%
High income ($189,000 or above)	21	3.6%
Insured (medical)		
No	87	14.8%
Yes	502	85.2%

The Qualtrics survey, conducted between July and December 2023, is provided in Supplementary Material 2. Eligible participants were aged 25–64, English proficient and residing in the U.S. They received $4 per submission ($13.33 per hour). The full survey included two attention-checks; 21 AMHL Index items; seven sociodemographic questions; and eight health-related outcome measures: mammography, colon cancer screening, COVID-19 and flu vaccination, annual exam attendance, smoking, vaping, and chronic condition diagnosis. A total of 603 participants completed the anonymous survey. Fourteen were removed for failing attention checks or straightlining, resulting in a final sample of 589, exceeding the required minimum.

### Statistical approach: external validation

First, regression analyses were conducted in R 4.4.2. to inspect variance inflation factors (VIF), a standard metric of collinearity. High multicollinearity—which is when items (“indicators”) are redundant and too strongly correlated with one another—can undermine the stability and interpretability of indicator weights in formative models, which are intended to comprise a range of items with distinct contributions. Unlike reflective measures,¶ formative indicators collectively form the construct rather than reflecting an underlying latent factor. Items are not intended to be interchangeable and removing one would theoretically alter the meaning and integrity of the whole.

Next, Partial Least Squares Structural Equation Modeling (PLS-SEM) was employed to establish nomological validity—i.e., whether scores on the Index significantly correlate with the outcomes of interest. PLS-SEM is the recommended statistical approach as it specifies indicators as direct causes of a composite score without requiring internal consistency or covariance (39, 44, 56–58).

Finally, covariate-adjusted linear and logistic regressions were conducted to test whether the Index would serve as an independent predictor after controlling for known co-variates. To account for interaction effects, multiplicative interaction terms were added to the logistic regression models to test whether associations between AMHL and the outcome variables were moderated by key demographic variables (education, race, age, gender, insurance).

## Results

### Score distribution

The highest score possible on the AMHL Index is 43 and the lowest is 0. Participants’ scores were calculated using the pre-established rubric (Supplementary Material 3). Scores were normally distributed as confirmed by a histogram and QQ plot. The sample mean was 30.3 (SD = 6.4) and scores ranged from 13 to 43.

### Multicollinearity checks

As a general guideline, VIF values below 10 are acceptable and values below 3.3 are considered excellent (59, 60). All 30 Index indicators** had VIF values considerably below 10, with 27 items below 3.3 (Table 2). There are no serious multicollinearity issues and the conceptual framework effectively captured each variable and the overarching AMHL construct. All indicators were retained for subsequent analysis.

**Table 2:** Indicator collinearity assessment, VIF results.

Domains	Indicator	VIF value
Access	HealthInfoSources	1.1
	EHR	1.1
	PCP	1.2
	MediaOrgs	1.3
	Journalists	1.4
	MedContentRegs	1.4
Identify and critically evaluate	Scenario1	1.3
	Scenario1A	1.1
	Deepfake	1.2
	Buzzfeed1	1.3
	Buzzfeed2	1.2
	Medline	1.1
	TikTok	1.1
	FreestoneReliable	2.1
	FreestoneObjective	2.3
	FreestoneAuthor	1.7
	FreestoneFU	3.3
	WorldView	1.2
	RaceView	1.3
Produce	TwitterProd	2.8
	InstaProd	3.6
	SnapchatProd	1.6
	TikTokProd	2.6
	RedditProd	3.4
	YelpProd	1.4
Engage	FacebookEng	1.2
	TwitterEng	2.7
	InstaEng	3.4
	TikTokEng	2.5
	RedditEng	3.4

### PLS-SEM: validating the hypothesized model

Using PLS-SEM, the model was further validated through an examination of strength and significance of path coefficients, and explained variance of outcome variables (R²). As shown in Table 3, results reflect the stability and relevance of indicator contributions. The structural model demonstrated the Index's ability to explain variance across all outcomes. While bootstrapping procedures were not feasible due to the binary character of the outcome variables, the theoretical consistency and stability of the model paths provide strong confidence in the external validity of the Index.

**Table 3:** Path coefficients and R² values.

Outcome	B^a^: path coefficient (AMHL → Outcome)	R²	Adjusted R²
Chronic health	0.2	0.0	0.0
Smoke	−0.3	0.1	0.1
Vape	−0.3	0.1	0.1
Mammogram	0.4	0.1	0.1
Colon screening	0.4	0.2	0.2
Covid vaccination	0.4	0.2	0.2
Flu vaccination	0.5	0.3	0.2
Annual exam	0.4	0.2	0.1

^a^ B values represent standardized path coefficients from PLS-SEM using sample data.

### Linear and logistic regressions: hypotheses results

Logistic regression models were run for each outcome, controlling for known covariates. As shown in Table 4, the overall findings show that one's composite score on the Index significantly predicted positive preventive behaviors in six of the seven health-behavior models, as well as in the final health-outcome model. The results validate the importance of adjusting for SDH and confirm AMHL's measurable impact on health decision-making.

**Table 4:** Logistic regression results (models a–g, adjusted).

Model	Variable	Odds Ratio	95% CI
Flu vaccination	(Intercept)	0.1	0.0–0.2***
	AMHLScore	1.1	1.0–1.1***
	Politics	0.7	0.6–0.9**
	Education	2.0	1.6–2.6***
	Age	1.0	1.0–1.0*
Covid vaccination	(Intercept)	0.0	0.0–0.1***
	AMHLScore	1.1	1.0–1.1***
	Politics	0.5	0.4–0.6***
	Education	2.2	1.7–2.9***
	Age	1.0	1.0–1.0**
Mammogram	(Intercept)	0.0	0.0–0.2**
	AMHLScore	1.1	1.0–1.1*
	Insurance	6.5	1.7–43.0*
	Race	1.2	0.7–2.2
Colon screen	(Intercept)	0.2	0.0–0.9*
	AMHLScore	1.0	1.0–1.1
	Insurance	3.0	1.0–11.0
	Race	0.7	0.4–1.2
Annual exam	(Intercept)	0.0	0.0–0.2***
	AMHLScore	1.0	1.0–1.1*
	Age	1.0	1.0–1.0**
	Insurance	8.3	4.9–14.5***
	Gender	1.0	0.7–1.5
Smoke	(Intercept)	1.1	0.3–3.5
	AMHLScore	0.9	0.9–1.0***
	Age	1.0	1.0–1.0*
	Education	1.0	0.7–1.3
	Race	1.0	0.7–1.3
Vape	(Intercept)	1.1	0.3–3.9
	AMHLScore	1.0	0.9–1.0**
	Age	1.0	1.0–1.0
	Education	1.3	1.0
	Race	0.7	0.4–1.0*
Chronic health	(Intercept)	0.0	0.0–0.1***
	AMHLScore	1.0	1.0–1.1*
	AnnualExam	2.0	1.4–3.0***
	Age	1.0	1.0–1.1***
	Gender	1.2	0.8–1.6

* *p* < .05.

** *p* < .01.

*** *p* < .001.

#### Flu vaccination

At the time of the survey in 2023, 40% of respondents reported receiving the latest influenza vaccine, comparable to federal estimates of 45% (61). In line with extant literature, those with more conservative political views had lower odds of flu vaccination (OR = 0.719, *p* = .003). Higher education substantially increased the likelihood of vaccination (OR = 1.988, *p* < .001), as did Age (OR = 1.020, *p* = .014). Controlling for these covariates, AMHL scores were significantly associated with increased odds of flu vaccination (OR = 1.051, *p* < .001), supporting H1a. This can be interpreted as a one-unit increase on the Index increases the odds of immunization by 5.1%.

#### COVID-19 vaccination

Thirty-nine percent of respondents in 2023 had received the most recent COVID-19 vaccine compared to 23% nationally for the 2024–2025 season (61). As with flu vaccination, significant covariates included Politics (OR = 0.500, *p* < .001), Education (OR = 2.202, *p* < .001), and Age (OR = 1.027, *p* = .002). Controlling for these covariates, AMHL score remained a strong independent predictor (OR = 1.082, *p* < .001), supporting H1b. Thus, a one-point increase in AMHL increases the odds of COVID-19 vaccination by 8.2%.

#### Mammography

Consistent with national data, 52% of women in the study have not received a recommended mammogram. Insurance strongly predicted screening (OR = 6.471, *p* = .018). Race was not a significant covariate, despite prior findings. Controlling for Insurance and Race, AMHL score remained a positive predictor (OR = 1.062, *p* = .040), supporting H1c. Each one-unit increase in AMHL was associated with a 6.2% increase in the odds of receiving a recommended mammogram.

#### Colon cancer screening

Similar to existing data, 55% of respondents had not received recommended colorectal screenings. After controlling for Insurance, which was marginally associated (OR = 3.021, *p* = .059), AMHL score was not a significant predictor (OR = 1.027, *p* = .203). Therefore, H1d was not supported.

#### Annual exam attendance

Age (OR = 1.027, *p* = .004) and especially Insurance (OR = 8.303, *p* < .001) showed strong effects. Controlling for these, AMHL score independently predicted a greater likelihood of annual check-up attendance (OR = 1.038, *p* = .018), with each one-unit increase linked to a 3.6% rise in odds—supporting H1e.

#### Smoking

About 25% of participants identified as cigarette smokers—10% higher than the most recent national estimates in 2022 (30). After controlling for Race, Education, and Age—which was the only co-variate that reached a statistically significant association (OR = 1.020, *p* = .020)—AMHL score remained an independent predictor of smoking (OR = 0.937, *p* < .001). Each one-unit increase lowers the odds of smoking by 6.3%, thereby affirming H2a.

#### Vaping

About 20% of participants reported vaping daily or occasionally—12% higher than 2023 estimates (29). Race was significantly associated (OR = 0.666, *p* = .042), with the highest rates among Black/African Americans and the lowest rates among Asian Americans. Research on racial differences in vaping is mixed, with some studies showing higher rates of usage among populations with higher cigarette smoking rates who adopt vaping as a cessation aid.

Controlling for Race, Education and Age, AMHL score was negatively associated with Vaping (OR = 0.953, *p* = .004), supporting H2b. Each one-unit increase in AMHL reduced vaping odds by 4.7%.

#### Chronic health condition

Annual Exam attendance was strongly associated with reporting a chronic health condition (OR = 2.001, *p* < .001), as were Age (OR = 1.042, *p* < .001) and Income (OR = 0.568, *p* = .002), consistent with known disparities in chronic illness prevalence. Controlling for these, a higher AMHL score was associated with increased odds of reporting a chronic health condition (OR = 1.037, *p* = .010). This contradicts H3, which theorized that AMHL would reduce chronic disease through greater preventive behaviors. While the results may appear counterintuitive, we offer several potential explanations. First, those with higher levels of AMHL may have greater diagnostic awareness, meaning they may be more likely to proactively access reliable health information and seek medical advice, resulting in higher rates of diagnosis. Inversely, those with chronic health conditions—particularly those that require active management, such as diabetes—likely have greater engagement with the health care system and increased motivation to learn about their condition, resulting in a higher level of AMHL. Finally, an overly broad categorization of “chronic diseases” in the survey prompt may have also contributed to this result as it failed to exclude or differentiate between more preventable chronic conditions and those associated with genetics or environmental factors beyond the individual's control.

#### Demographic interaction effects

No significant interactions were found for most demographics. Notable exceptions included education, which moderated the association between AMHL and smoking (interaction ORs for Bachelor's and Master's/Doctorate vs. no four-year degree = 0.90, *p* < 0.001) and vaping (interaction ORs = 0.90, *p* < 0.001), indicating that AMHL's protective effect against smoking and vaping was stronger among participants without a four-year degree. Race also moderated the association between AMHL and COVID-19 vaccination (interaction OR = 0.91 for Black or African American vs. white, *p* = 0.017; interaction OR = 1.64 for American Indian or Alaska Native vs. White, *p* = 0.046), with AMHL having a weaker effect among Black or African American participants and a stronger effect among American Indian or Alaska Native participants compared to white participants.

#### Additional insights

Performance on individual Index items also offers insight for intervention through a more granular inspection of AMHL. In line with research that shows growing reliance on digital media, 72% said they “get most of their basic health information”^††^ from the internet (“I Google it”) and social media. Only 20% chose their personal doctors, which aligns with decreasing reliance on HCPs for non-acute care and health information (62). This is notable when considering that 38% were unable to identify the key job functions of a primary care doctor, such as referring patients to specialists and prescribing medications.

Several Index items—including scenario prompts, social media posts and videos, and “sponsored” posts—probed respondents’ abilities to identify key elements of message construction and quality, as well as knowledge of media processes. For example, only 40% knew U.S. journalists are not required to be licensed or field trained. One third also falsely believe (or might believe: “Not sure”) that “there are regulations that require all medical information posted on the internet to be reviewed by a medical professional before it is posted.” When seeking reliable health information, 16% chose “the first result on Google” as a top indicator of source reliability and 30% deemed MedlinePlus.gov—visibility promoted as a service of the NIH—as an unreliable source for health information.

Participants also reviewed screenshots of content from a fictitious source called the Freestone Institute, which was modeled after the Brownstone Institute - a special-interest group criticized by the scientific community for promoting discredited health misinformation (77–82). A dummy Twitter account (now X) and AI-generated images were produced to prevent respondents' Googling the source while responding.

**Figure 3:**
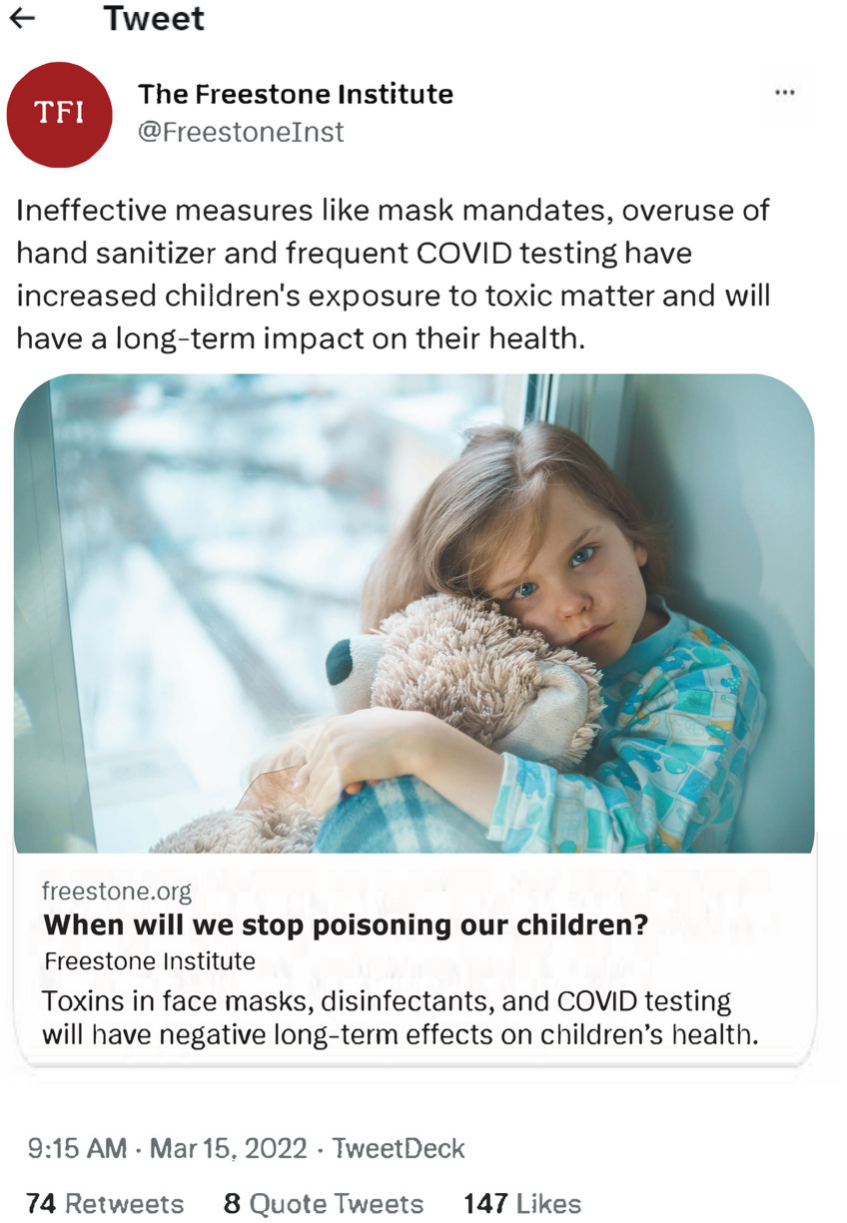
Freestone Tweet for appraisal.

As shown in Figure 3, they were first asked to appraise the content of a Tweet posted by the Freestone Institute, which 23% incorrectly rated as “reliable.” They were then given more background information on the source:

Founded in 2021, the Freestone Institute is a nonprofit think tank that focuses on public health and economic research. We believe in a society that places an individual's freedom over government mandates. We employ a small, hardworking team with no bureaucrats and we rely on outside help from intellectuals, scientists and others. The Freestone Institute focuses on op-eds, analysis and research. We are not a news organization. Fact-checking and content production are the responsibility of the authors.

**Figure 4:**
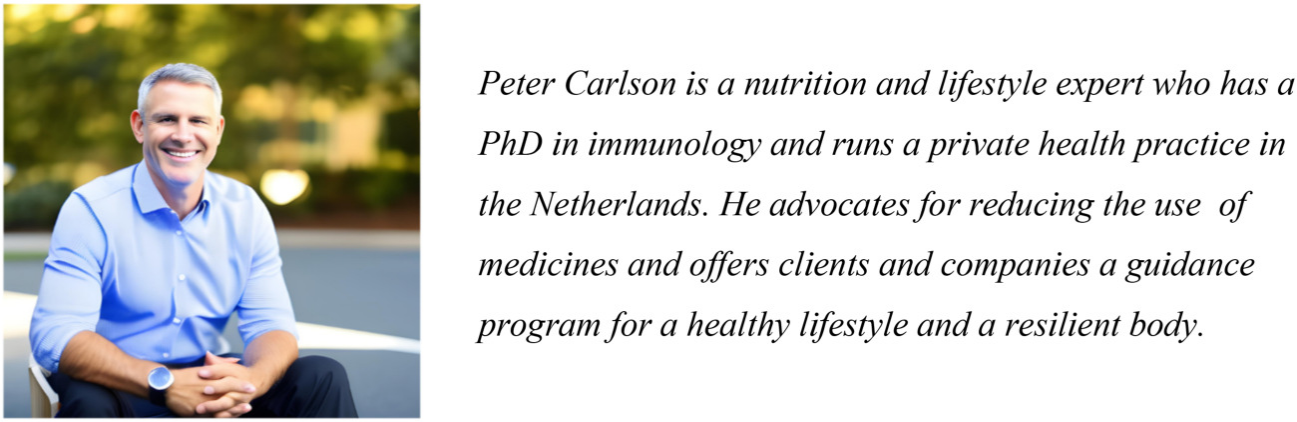
Description and photo of article's author.

One-third then rated the Freestone Institute as an “objective source.” Next, participants were shown the photo and bio of the author who wrote the article (Figure 4), and 62% rated him as “qualified to write about *children's* health.” Finally, they re-evaluated the original Tweet with the additional information they had received. Notably, the percentage of respondents who viewed the original post as “reliable” *increased* from 18% to 41%, despite each new piece of information revealing implicit and explicit biases. These findings highlight the widespread need for greater MHL among adults—especially amid an information ecosystem that is increasingly rife with insidious content promoted by deceptive, self-appointed think tanks and “experts.”

These results shed light on the mean Index score of 30 (equivalent to a C-), which may in part reflect two sociopolitical factors. First, the majority of adults in the U.S. have not received any media education throughout their lifetimes (63). In fact, the U.S. ranks 17th among all democratic countries on the Open Society Institute's Media Literacy Index, which measures nations’ vulnerability to disinformation (63). In addition, the U.S. is the only industrialized nation without universal health insurance and where roughly 88% of adults lack proficient HL skills, such as interpreting prescription labels and understanding the purpose of preventive care (64, 65). Taken together, these structural deficits help contextualize the study's results and inform future research directions.

## Discussion

Situated at the intersection of public health and communication studies, this mixed-methods study expands our understanding of ML's relationship with human health. Methodologically, it introduces the Adult Media Health Literacy Index, the first validated measurement of AMHL. Conceptually, the findings support the expansion of socio-ecological health models to include adult ML as an independent social determinant of health. Correlational analyses confirmed six of the eight hypotheses and demonstrated that the AMHL Index is a reliable and independent predictor of health behaviors.

A *single point* increase on the AMHL Index was found to be associated with an 8.2% increase in the odds of receiving the COVID-19 vaccination, a 6.2% increase in the odds of undergoing a mammogram, and a 3.6% increase in the odds of attending an annual medical exam. On risk mitigation, a one-point increase on the Index was shown to lower the odds of smoking by 6.3% and the odds of vaping by 4.7%. To contextualize the results more broadly, odds changes of 5%–10% in public health and behavioral research are considered meaningful, particularly when they are tied to modifiable factors like AMHL.

The findings also suggest that the impact of AMHL on certain preventive or risky behaviors may vary by education or race, though the majority of outcomes did not show significant moderation. As it pertains to smoking and vaping, the stronger effect among groups with lower levels of education is consistent with previous research and could indicate a greater marginal benefit of AMHL in populations with fewer formal educational resources. In regard to the moderating role of race on the likelihood of COVID-19 vaccination, the findings are in line with national trend reports on vaccination coverage. Such disparities have been linked to social and structural factors, such as insufficient health care access or greater vaccine hesitancy as a result of racial/ethnic discrimination (66–68).

Collectively, the results are not only statistically significant but practically important for health communication, media education and public policy interventions.

## Public health implications

Amid budget cuts to public health infrastructure and an unregulated digital media landscape, the American public's exposure to health mis/disinformation will continue to increase while their access to essential health resources decrease (e.g., Medicaid cuts, vaccine rollbacks, and water-fluoridation bans). Investing in multi-sector interventions that can engender greater MHL across the lifespan can contribute to the mitigation of preventable diseases, with spillover effects to the types of informed decision-making that buoy healthy democracies. While specific proposals are beyond the scope of this study, opportunities include:

• Integrating media literacy into CDC's health promotion strategy;
• Institutionalizing media education across the lifespan (69);
• Implementing evidence-based technology interventions, such as digital “nudging,” pre- and debunking, automated content labeling, and algorithmic restructuring (70);
• Enacting consumer protection legislation (71, 72);
• Exploring alternative revenue models that reduce financial incentives associated with the spread of mis/disinformation (73, 74); and
• Integrating misinformation-specific communication training in medical school and CME curricula for trainees and health care professionals (75).

As with most public health efforts, a socio-ecological model is often best suited to understand and address complex challenges that require multi-level intervention. The mitigation of health mis/disinformation is one of those challenges and requires a re-imagination of the social determinants that underpin human health in modern society, chief among them—media literacy.

### Limitations

This study employed a non-probability sampling method, which may limit the generalizability of the findings and potentially introduce selection bias and affect the external validity of the results. However, the use of quota-based sampling helped minimize these limitations. Given that the sample was closely matched to national demographic data for age, gender, race, and education—and in light of minimal demographic deviations—we elected not to weight the data. This decision simplified interpretability and preserved analytical clarity, though it is acknowledged as a potential limitation.

### Future research

For explanatory purposes, future studies should include additional health measures—such as sunscreen usage, reproductive health, diet and physical activity—as well as theoretically-guided mediation analyses. Given the dynamic nature of MHL across the lifespan, longitudinal validation would also illuminate causal relationships and fluctuation by age. In addition, adapting the Index for use in countries with universal health care or robust public media systems may reveal how AMHL manifests in less commercialized contexts and provide insights into the adaptability of the tool across diverse populations and public health systems. Research on pragmatic short- and long-term interventions is also urgently needed.

## Data Availability

The raw data supporting the conclusions of this article will be made available by the authors, without undue reservation.

## Footnotes

*Misinformation is defined as information that is false or misleading according to the best available evidence at the time (43). Misinformation falls on a wide spectrum, from decidedly inaccurate (e.g., all flu vaccines contain live viruses) to partially true (e.g., natural immunity is better than vaccine immunity) to more nuanced or context-specific grey areas (e.g., depression is caused by a chemical imbalance). Disinformation is the intentional production and dissemination of false information.

^†^Defined as a health and wellness tool, resource, trend or product (24)

^‡^The term “media literacy” is used as an umbrella construct and includes media health literacy, information literacy, digital literacy, news ML, and critical ML, among others.

^§^While some conditions, such as lung cancer, are more often associated with behavioral factors such as smoking, others are linked to genetic and hereditary predispositions, making them less “preventable” but highly treatable if detected early. The term preventable refers to conditions that can be all together avoided, as well as the prevention of premature mortality associated with forgone measures such as screenings and vaccination.

^||^These are often collapsed to micro-, meso- and macrosystems. The “chronological” level refers to the life course and is central to understanding AMHL as a dynamic literacy that fluctuates over time.

^¶^Common examples of reflective constructs include scales that measure attitudes, beliefs or perceptions.

^**^The Index includes 21 questions, two of which are designed as a matrix; hence, the 30 indicators.

^††^"Health information" was defined as information about health, such as information about diseases/conditions, medicines, vaccines, nutrition, mental health, etc.
