# Supplementary material for: Media health literacy predicts preventive health behaviors: findings from a nationally matched survey
Source: Front Digit Health. 2025 Sep 30;7:1659988. doi: 10.3389/fdgth.2025.1659988 (PMC12518239; doi:10.3389/fdgth.2025.1659988)
Supplement: Supplementary file 1 [file Datasheet1.pdf]

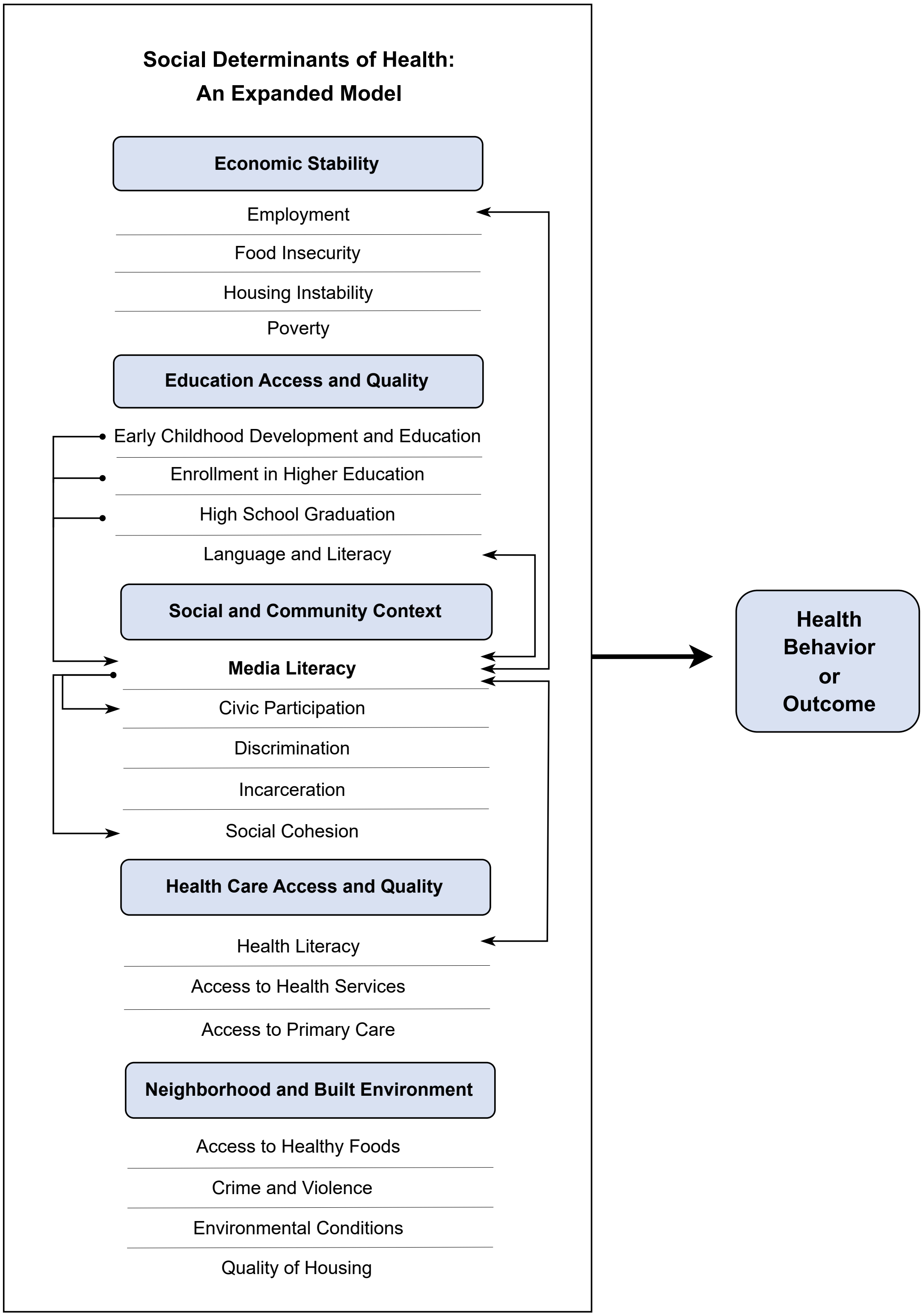

Citation:
Ashtari, S. (2025, February 24). *Media health literacy: A scoping review and agenda for future research*. *International Journal of Communication, 19*, 1228–1251. https://ijoc.org/index.php/ijoc/article/view/23798
