# Supplementary material for: Media health literacy predicts preventive health behaviors: findings from a nationally matched survey
Source: Front Digit Health. 2025 Sep 30;7:1659988. doi: 10.3389/fdgth.2025.1659988 (PMC12518239; doi:10.3389/fdgth.2025.1659988)
Supplement: Supplementary file 2 [file Datasheet2.pdf]

Adult Media Health Literacy: Definition and Competencies

DEFINITION

Adult media health literacy (AMHL) is a cumulative competency that requires five core abilities: (1) ***access*** health-related information; (2) ***identify*** key elements of content construction; (3) ***critically evaluate*** content credibility, quality and relevance; (4) ***produce*** content using a variety of media tools; and (5) ***engage*** with a global media culture.

Each component is operationalized as follows:

(1) ***access*** health-related information.

- **a)** Recognizes when health information is needed.
- **b)** Has ability to locate the appropriate information from a *variety of sources* and can functionally understand the information at hand.
- **c)** Possesses enough functional, scientific and information literacy to understand the meaning of common yet important health- *and* media-related words, processes, roles, symbols and techniques.
- **d)** Can access health provider's online electronic medical records to view personal health information.

(2) ***identify*** key elements of content construction.

- **a)** Identifies key media-construction concepts, such as authorship (who crafted the content), audience (who is intended to see it) and creative techniques (e.g., point of view or format).
- **b)** Understands the implications of different content formats (e.g., editorial vs academic research article).
- **c)** Knows that algorithms play a role in what we see online.

(3) ***critically evaluate*** content credibility, quality and representativeness based on its source, substance and form.

- **a)** Recognizes explicit and implicit message points.
- **b)** Recognizes the interests of content producers (e.g., commercial vs noncommercial) and how they might shape the message.

c) Uses various critical-reasoning strategies – such as fact-checking, compare/contrast, identifying bias and recognizing conflict of interest – to judge credibility and quality.

d) Understands the implications of anecdotal vs empirical evidence.

e) Examines multiple sources and/or solicits input when seeking health information and making health-related decisions.

f) Can identify which values, points of view and lifestyles are included and omitted from a message.

g) Recognizes visual/narrative representation as a powerful social and political tool.

h) Recognizes that their interpretation of media messages is shaped by their life experiences, culture, social networks, socioeconomic position and identity.

(4) ***produce*** content using a variety of media and digital tools.

Can create content in various formats (social media text posts, social media video posts, web content, emails, video production, podcasting, blogging, Google docs, etc.).

(5) ***engage*** with a global media culture.

Utilizes various media resources to gain greater understanding of an issue, contribute to a knowledge pool and/or form a sense of community (e.g., engagement with posts on social aggregation sites like Quora and Reddit, reviews on crowdsourcing sites such as Yelp or Nextdoor, engagement with online marketplaces like Craigslist, dating apps/sites, letters to the Editor, radio call-ins, sharing others' posts on social media, etc.).

Notes:

The definition provided comprises a cumulative set of competencies that determine an adult's level of media health literacy. A key point here is that AMHL is not a dichotomous skill or ability; individuals fall on a spectrum and levels of AMHL can and do fluctuate throughout the life course.

In addition, while the competencies listed above generally build on each other, this definition does not assume that AMHL competencies are necessarily progressive in nature, meaning individuals may have some capacity to identify key media-construction concepts (AMHL #3) but may not be able to produce content using contemporary media tools (AMHL #2).

To learn more about the development and operationalization of AMHL, see:
Ashtari, S. (2025). Media health literacy: A scoping review and agenda for future research. International Journal of Communication, 19, 1228–1251. Retrieved from https://ijoc.org/index.php/ijoc/article/view/23798

To measure AMHL, contact sashtari@usc.edu for a copy of the AMHL Index, a validated performance-based measurement.
