# Supplementary material for: Media health literacy predicts preventive health behaviors: findings from a nationally matched survey
Source: Front Digit Health. 2025 Sep 30;7:1659988. doi: 10.3389/fdgth.2025.1659988 (PMC12518239; doi:10.3389/fdgth.2025.1659988)
Supplement: Supplementary file 3 [file Datasheet3.pdf]

Adult Media Health Literacy Index

Administrator Instructions

- The Adult Media Health Literacy Index is designed for adults between the ages of 18 and 64 who are fluent in English. It was last validated in March 2025.
- It should be administered online. Access to internet resources during the assessment should be disabled or, if not possible, strongly discouraged.
- No more than 30 minutes should be allotted for each submission and it should be taken continuously (in one sitting). The average time for completion is 10 - 15 minutes.
- Stimuli should not include live links; i.e., participants should view screenshots or links to internally hosted, static images/videos so as not to expose them to or direct traffic to websites or social media sources with dangerous health mis/disinformation. Stimuli are available on the Open Science Framework and linked below. Survey administrators should embed images and video directly in the survey.
- A fact-check debrief should be shared with participants after they submit the assessment to counteract their exposure to the mis/disinformation presented during the assessment.
- The full survey is provided on the following pages. The Index comprises 21 questions – Q4 - Q6; Q8 - Q14; and Q16 - Q26 – and they should be programmed on Qualtrics as forced responses.
- Scoring: Correct answers on knowledge questions are bolded. **Scoring/point allotments** for the Index are annotated throughout in **red** (for administrators) and a score sheet is provided at the end of this document. Automated coding/scoring in Qualtrics/Excel is recommended.
- Optional questions (*not* part of the Index): The survey includes two attention-check questions (Q7 and Q15); eight health behavior/outcomes questions (Q28 - Q35), and nine socio-demographic (Q1, Q3, Q27 and Q36-Q41). These are not part of the Adult MHL Index. Include at your discretion and as desired for research purposes. **Coding** (not scoring) of these non-Index items are noted in **blue.**
- Square response boxes indicate multiple selections. Circular response buttons indicate single-answer response choices.

START

< Informed consent portion excluded for length >

---

1. Please enter your age in years: _____________

2. Are you comfortable reading *and* speaking in English?

- ○ Yes
- ○ No

3. What is your current gender identity?

- ○ Man
- ○ Woman
- ○ Gender queer
- ○ Gender non-binary/gender non-conforming

<If passed screeners>: Let's get started!

The first few questions will ask about how you find and use **health information**. "Health information" refers to ***information about health***, such as information about diseases/conditions, medicines, vaccines, nutrition, mental health and other topics related to your health. In this survey, "health information" is <u>not</u> referring to health insurance.

4. Where do you get most of your basic health information?

- ○ Television news 1
- ○ My doctor(s) 2
- ○ The internet (I Google it) 1
- ○ Friends and family who are medical professionals 2
- ○ Friends and family who are not medical professionals 1
- ○ Social media (such as Facebook, TikTok, Twitter, etc.) 0

Points: ☐ 0 ☐ 1 ☐ 2

The next section is going to describe a pretend scenario and ask you questions based on that scenario.

5. Imagine you come home one day and you feel sick. You have sharp stomach pains, a fever and you're vomiting. After three days, you're only feeling worse so you start searching the internet for more information. Your internet search is showing you information from the five sources listed below.

Please select THREE sources that you think will offer you the most reliable health information in this scenario:

- ☐ A personal blog by a doctor
- ☐ **A patient education webpage by the Mayo Clinic, a national nonprofit medical center**
- ☐ An article by a journalist at a news agency (such as CNN)
- ☐ **The National Institutes of Health (NIH) website, which publishes research funded by the U.S. government**
- ☐ The first result at the top of the Google results page
- ☐ **A health-information website that is reviewed by medical experts (such as Healthline)**

0 = Only one right answer among selections
1 = One right answer among selections
2 = Two right answers among selections
3 = All three correct answers selected

Points: ☐ 0 ☐ 1 ☐ 2 ☐ 3

6. Now imagine two of the sources you chose above have very different advice on what you should do about your symptoms. Which of the following options *most closely* describes **how you would decide between the two conflicting pieces of information?**

- ○ I would not follow either piece of advice and would tolerate my symptoms for a while longer 0
- ○ I would pick the advice that matches what I already believe 0
- ○ I would ask a friend or family member for their opinion 0
- ○ I would reach out to a doctor or other health care provider 1
- ○ I would do more online research to figure out which source is more scientifically credible and trustworthy 1

Points: ☐ 0 ☐ 1

7. The TV show question you are about to answer is very simple. When asked for your favorite TV show, you must select "Law & Order." This is an attention check.

- ○ Euphoria
- ○ **Law & Order**
- ○ Succession
- ○ Friends
- ○ West Wing

Remember to read and answer questions carefully and honestly. Let's continue!

The next two questions are going to ask you about different health care terms.

---

8. Your doctor's office may use an online website/portal to store your medical information, such as visit summaries or results from a blood test. These are often referred to as your "electronic health records."

Do you know how to access and view your electronic health records?

- ○ Yes 1
- ○ No 0
- ○ Not sure 0

Points: ☐ **0** ☐ **1**

9. Typically, a **primary care doctor** (sometimes called a "general physician" or a "family doctor") is responsible for which of the following? **Select all that apply.**

- ☐ **Prescribing medications as needed**
- ☐ **Overseeing all basic aspects of your health care**
- ☐ **Referring you to specialists**
- ☐ Conducting emergency surgeries
- ☐ **Diagnosing a wide variety of medical conditions**
- ☐ Being immediately available when you have a life-threatening emergency
- ☐ **Conducting preventive tests and physical exams**

2 = Selects all and only the five correct options
1 = Selects four of the five correct answers and *no others*.
0 = All others

Points: ☐ **0** ☐ **1** ☐ **2**

The next few questions will ask you about the media in the United States, such as television, online news and movies. For each question, please select the single best answer.

10. In the United States, the majority of media organizations (such as Warner Brothers, CNN, NBC, Fox, etc.) are:

- ○ financially supported by the government through public taxes 0
- ○ **for-profit businesses 1**
- ○ non-profit businesses 0
- ○ owned by companies outside the United States 0
- ○ financially supported by religious organizations 0

Points: ☐ **0** ☐ **1**

11. To work as a professional news reporter in the United States, journalists are:

- ○ required to obtain a professional license from the Federal Communications Commission (FCC) 0
- ○ required to obtain a professional license from the Society of Professional Journalists 0
- ○ required to complete five years of field training 0
- ○ **not required to be licensed 1**
- ○ required to give up their right to vote in U.S. elections 0

Points: ☐ **0** ☐ **1**

12. There are regulations that require all medical information posted online to be reviewed by a medical professional before it is posted.

- ○ True 0
- ○ **False 2**
- ○ Not sure 0

Points: ☐ **0** ☐ **2**

The next two questions are going to ask you about your engagement with different types of media and media technology.

13. Do you know how to **create** your own messages, posts or content on the following platforms?

	Yes	No
**a.** Twitter	○ 1	○ 0
**b.** Instagram	○ 1	○ 0
**c.** Snapchat	○ 1	○ 0
**d.** TikTok	○ 1	○ 0
**e.** Reddit	○ 1	○ 0
**f.** Yelp	○ 1	○ 0

Points: ☐ **0** ☐ **1** ☐ **2** ☐ **3** ☐ **4** ☐ **5** ☐ **6**

14. Do you know how to **engage** with messages, posts or content on the following platforms?
Examples of "engagement" include "liking," sharing or commenting on someone's post.

	Yes	No
**a.** Facebook	○ 1	○ 0
**b.** Twitter	○ 1	○ 0
**c.** Instagram	○ 1	○ 0
**d.** TikTok	○ 1	○ 0
**e.** Reddit	○ 1	○ 0

Points: ☐ **0** ☐ **1** ☐ **2** ☐ **3** ☐ **4** ☐ **5**

15. Please select "Strongly agree" as your answer to this question. This is an attention check.

- ○ Strongly disagree
- ○ Disagree
- ○ Somewhat disagree
- ○ Somewhat agree
- ○ Agree
- ○ **Strongly agree**

You're doing great and you're nearly halfway through!

The following section will show you different media content and ask you questions about them.

Please watch this short video with your audio ON and then answer the question that follows:

< *For survey administrator:* **stimuli LINK - Zuckerberg video** >

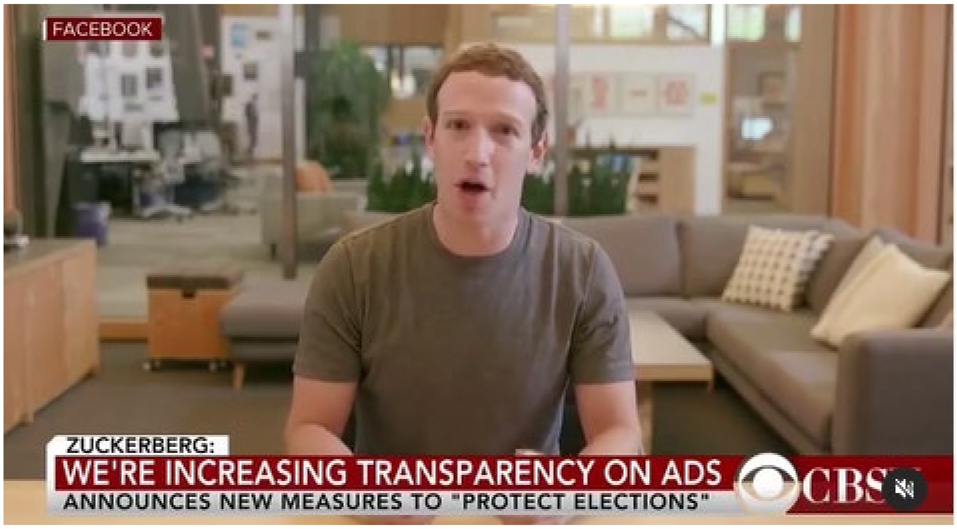

16. Please choose the single best answer.

The video above:

- ○ Was probably leaked by someone at Facebook/Meta 0
- ○ Confirms what I already believed 0
- ○ Contradicts what I believed 0
- ○ **Seems like a fake video** 1
- ○ Makes me feel angry with Mark Zuckerberg 0

Points: ☐ 0 ☐ 1

Below you will see a link to a **BuzzFeed post** titled "25 Songs Guaranteed To Give You The Munchies." After you click the link below, it will open in a new tab. From there, you will be able to click and zoom in, if needed.

Spend up to 1 minute skimming the content then return to this survey and answer the questions that follow. **Please** click here **to open the image we'd like you to review.**
< *For survey administrator:* **stimuli LINK - Buzzfeed article** >

---

17. Which answer below best characterizes the BuzzFeed post above?

- ○ A meme 0
- ○ A personal blog 0
- ○ A news article 0
- ○ **An advertisement 2**
- ○ An infographic 0
- ○ An op-ed 0

Points: ☐ 0 ☐ 2

18. What do you think is the *main purpose* of the BuzzFeed post?

- ○ To promote 4/20 (a day of celebrating marijuana) as a nationally acceptable holiday 0
- ○ To generate website traffic for Buzzfeed ahead of 4/20 0
- ○ To sell marijuana products ahead of 4/20 0
- ○ To promote certain musical artists 0
- ○ **To promote Spotify, a music streaming service 2**

Points: ☐ **0** ☐ **2**

Below you will see a link to a website called **MedlinePlus.gov**. After you click the link below, it will open in a new tab. (You will be able to click it to zoom in, if you need to.) Spend up to 1 minute skimming the content then return to this survey and answer the question that follows. Please click here to open the image we'd like you to review.
< *For survey administrator:* **stimuli LINK - Medline Page** >

19. How reliable would you rate the information posted on MedlinePlus.gov?

- ○ Very unreliable 0
- ○ Unreliable 0
- ○ Somewhat unreliable 0
- ○ Somewhat reliable 1
- ○ Reliable 2
- ○ Very reliable 2

Points: ☐ **0** ☐ **1** ☐ **2**

Please view this short TikTok video before answering the question that follows:

< *For survey administrator:* **stimuli LINK - TikTok video** >

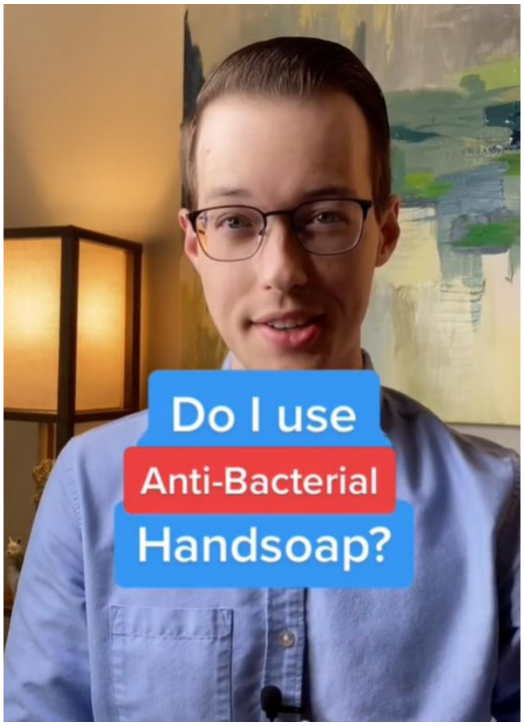

---

20. In the TikTok video you just watched, the man is sharing information about handwashing and anti-bacterial soap. Please select the **two most persuasive parts** of the man's video:

- ☐ He demonstrates how to wash your hands.
- ☐ **He tells us that he is a scientist.**
- ☐ He uses TikTok so most people can access it.
- ☐ **He cites data by the Food and Drug Administration (FDA).**
- ☐ He dresses professionally.

0 = Selects neither correct answer
1 = Selects one of the correct answers.
2 = Selects only the two correct answers.

Points: ☐ **0** ☐ **1** ☐ **2**

The next section is going to show you a Twitter post and ask you some questions about it.

Imagine you're scrolling through Twitter and you see the tweet below. Please take a moment to review the image of the tweet before answering the question below.
< *For survey administrator:* **stimuli LINK - Twitter post dummy** >

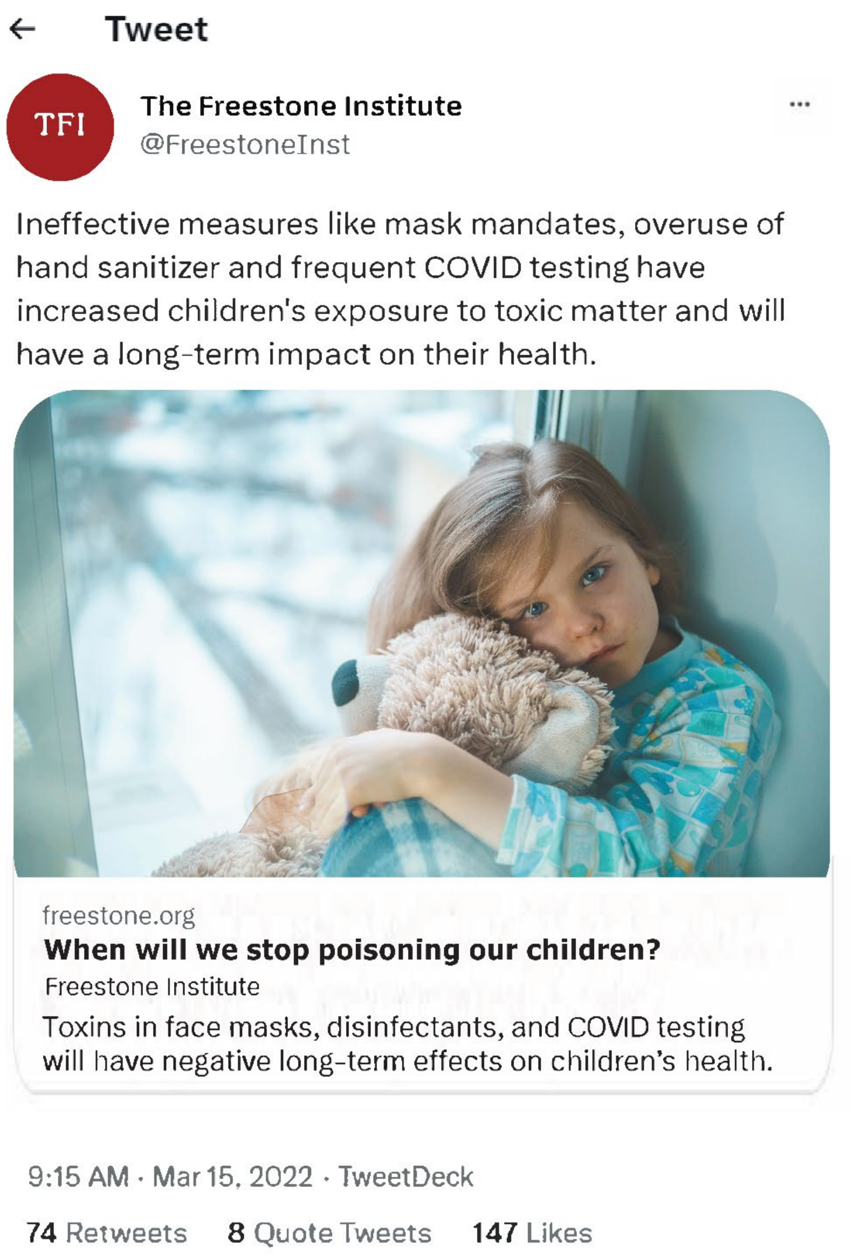

21. Please rate your level of agreement with the following statement:
The information in the tweet seems reliable.

- ○ Strongly disagree 2
- ○ Disagree 2
- ○ Somewhat disagree 1
- ○ Somewhat agree 0
- ○ Agree 0
- ○ Strongly agree 0

Points: ☐ **0** ☐ **1** ☐ **2**

You then notice that the article is posted by the Freestone Institute. Let's assume you have never heard of the Freestone Institute so you look at their website to learn more and you read the following:

22. Please rate your level of agreement with the following statement:
The Freestone Institute seems like an objective source for information.

- ○ Strongly disagree 2
- ○ Disagree 2
- ○ Somewhat disagree 1
- ○ Somewhat agree 0
- ○ Agree 0
- ○ Strongly agree 0

Points: ☐ **0** ☐ **1** ☐ **2**

In this same scenario, imagine you notice that the person who wrote the article for the Freestone Institute is tagged in the tweet. So you click the author's profile and see a little more information about him:
< *For survey administrator:* stimuli LINK - AI generated image >

"Peter Carlson is a nutrition and lifestyle expert who has a PhD in immunology and runs a private health practice in the Netherlands. He advocates for reducing the use of medicines and offers clients and companies a guidance program for a healthy lifestyle and a resilient body."

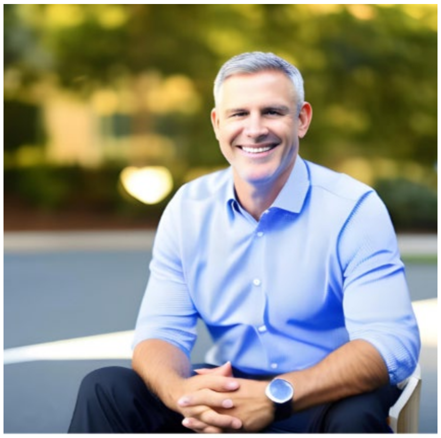

---

23. Please rate your level of agreement with the following statement:
The author of the article appears qualified to write about children's health.

- ○ Strongly disagree 2
- ○ Disagree 2
- ○ Somewhat disagree 1
- ○ Somewhat agree 0
- ○ Agree 0
- ○ Strongly agree 0

Points: ☐ **0** ☐ **1** ☐ **2**

24. Now that you have read a little more information, please rate your level of agreement with the following statement:
The information in the tweet posted by the Freestone Institute seems reliable.

- ○ Strongly disagree 2
- ○ Disagree 2
- ○ Somewhat disagree 1
- ○ Somewhat agree 0
- ○ Agree 0
- ○ Strongly agree 0

Points: ☐ **0** ☐ **1** ☐ **2**

The next two questions will ask you about your media habits and opinions more broadly.

---

25. Rate your level of agreement with the following statement:
My understanding of the world is shaped by the media I consume.

- ○ Strongly disagree 0
- ○ Disagree 0
- ○ Somewhat disagree 0
- ○ Somewhat agree 1
- ○ Agree 1
- ○ Strongly agree 1

Points: ☐ **0** ☐ **1**

26. Rate your level of agreement with the following statement:
My race/ethnicity influence how I interpret what I hear and see on TV/social media.

- ○ Strongly disagree 0
- ○ Disagree 0
- ○ Somewhat disagree 0
- ○ Somewhat agree 1
- ○ Agree 1
- ○ Strongly agree 1

Points: ☐ **0** ☐ **1**

This next set of questions will ask you about your health practices and health care experiences. (Reminder: All information in this survey is kept completely confidential.)

27. In the last three years, have you personally received harmful health advice from a health care professional (such as a doctor or nurse) that caused you pain, illness or disability?

- ○ No 0
- ○ Yes 1
- ○ Yes but not in the past three years 2

28. How often do you smoke cigarettes?

- ○ Every day 1
- ○ Some days 1
- ○ Never 0

29. How often do you smoke e-cigarettes (also called "vaping")?

- ○ Every day 1
- ○ Some days 1
- ○ Never 0

30. Have you ever been diagnosed with a chronic health condition? Examples include: a physical disability, high blood pressure, cancer, diabetes, lung disease, COPD, kidney disease, etc.

- ○ Yes 1
- ○ No 0

31. In the past 12 months, did you receive the flu vaccine?

- ○ Yes 1
- ○ No 0
- ○ I don't know 0

32. Have you received at least three doses of the COVID-19 vaccine?

- ○ Yes 1
- ○ No 0
- ○ I don't know 0

33. Have you taken any tests to check for colon cancer? (This can be an at-home stool test or a colon-cancer screening with your doctor.)
*This question is only included for participants who are men and 45 years old or above.

- ○ Yes 1
- ○ No 0
- ○ Not yet but it is scheduled 0

34. Have you received a mammogram in the <u>last 12 months</u>? (A mammogram uses an X-ray image to check for early signs of breast cancer.)
*This question is only included for participants who are women and 44 years old or above.

- ○ Yes 1
- ○ No 0
- ○ Not yet but it is scheduled 0

The next set of questions will ask you about your views on **routine annual exams** with your doctor. These routine check-ups are recommended once a year (or once every two years for younger adults). Sometimes these appointments are called "wellness visits" or "physicals." During these exams, your doctor checks your overall health and wellness by measuring your weight, height, blood pressure, heart rate and other routine measurements.

---

35. Have you seen your doctor for an annual exam in the <u>past 24 months</u>?

- ○ Yes 1
- ○ No 0
- ○ Not yet but it's scheduled 0

You're so close to the finish line! This final set of questions gives us basic demographic information. As a reminder, all of your answers are confidential.

---

36. What race do you identify with?

- American Indian or Alaska Native 3
- Asian 2
- Black or African American 0
- Native Hawaiian or Other Pacific Islander 2
- White 1
- More than one race 3
- Not listed: ______________________________________

37. Are you of Hispanic, Latino or Spanish origin?

- No 0
- Yes – I am Mexican, Mexican American, and/or Chicano 1
- Yes – I am Puerto Rican 1
- Yes – I am Cuban 1
- Yes – but of a different origin (for example, Salvadoran or Dominican): ____________ 1

38. What is the highest level of education you have reached?

- Did not finish high school / did not receive diploma 0
- Graduated from high school or got GED 0
- Some college, no degree 0
- Associate's degree 0
- Received a bachelor's degree 1
- Received a master's degree or equivalent 2
- Received a doctorate degree (PhD, MD or JD) 2

39. Of the options below, which best describes your current political affiliation?

- I lean toward Democrat 0
- I identify as Democrat 0
- I identify as politically Independent 2
- I identify as Republican 1
- I lean toward Republican 1
- I don't identify with any political affiliation 2

40. Do you currently have health insurance?

- Yes 1
- No 0

41. My annual household income is in the following category:

- $0 – $20,999 0
- $21,000-$41,999 0
- $42,000-$62,999 1
- $63,000-$83,999 1
- $84,000-$104,999 1
- $105,000-$125,999 1
- $126,000-$146,999 1
- $147,000-$167,999 2
- $168,000-$188,999 2
- $189,000 or above 2

You're done! Thank you for your time and thoughtful participation!

### END SURVEY ###

Adult MHL Index Scoresheet

Index Item Numbers*	Max pts possible	Points achieved
**Q4**	2	
**Q5**	3	
**Q6**	1	
**Q8**	1	
**Q9**	2	
**Q10**	1	
**Q11**	1	
**Q12**	2	
**Q13**	6	
**Q14**	5	
**Q16**	1	
**Q17**	2	
**Q18**	2	
**Q19**	2	
**Q20**	2	
**Q21**	2	
**Q22**	2	
**Q23**	2	
**Q24**	2	
**Q25**	1	
**Q26**	1	
	**TOTAL:**	/ 43 pts

**Proficiency Tiers**	
☐ No proficiency	13 - 17 points**
☐ Low proficiency	18 - 23 points
☐ Proficient	25 - 35 points
☐ High proficiency	36 - 43 points

**Numbered as they appear on Adult MHL Survey.*
***Scores below 13 should be examined for eligibility/completeness as these are extreme outliers.*
